# Severe Maternal Morbidity and Mortality Among Immigrant and Canadian-Born Women Residing Within Low-Income Neighborhoods in Ontario, Canada

**DOI:** 10.1001/jamanetworkopen.2022.56203

**Published:** 2023-02-16

**Authors:** Jennifer A. Jairam, Simone N. Vigod, Arjumand Siddiqi, Jun Guan, Alexa Boblitz, Xuesong Wang, Patricia O’Campo, Joel G. Ray

**Affiliations:** 1 Division of Epidemiology, Dalla Lana School of Public Health, University of Toronto, Toronto, Ontario, Canada; 2 MAP Centre for Urban Health Solutions, St Michael’s Hospital, Toronto, Ontario, Canada; 3 Department of Psychiatry, University of Toronto, Toronto, Ontario, Canada; 4 Institute of Health Policy, Management and Evaluation, University of Toronto, Toronto, Ontario, Canada; 5 ICES, Toronto, Ontario, Canada; 6 Department of Psychiatry, Women’s College Hospital, Toronto, Ontario, Canada; 7 Gillings School of Global Public Health, University of North Carolina-Chapel Hill, Chapel Hill; 8 Keenan Research Centre, St Michael’s Hospital, Toronto, Ontario, Canada; 9 Department of Obstetrics and Gynaecology, St Michael’s Hospital, Toronto, Ontario, Canada

## Abstract

**Importance:**

Evidence indicates that immigrant women and women residing within low-income neighborhoods experience higher adversity during pregnancy. Little is known about the comparative risk of severe maternal morbidity or mortality (SMM-M) among immigrant vs nonimmigrant women living in low-income areas.

**Objective:**

To compare the risk of SMM-M between immigrant and nonimmigrant women residing exclusively within low-income neighborhoods in Ontario, Canada.

**Design, Setting, and Participants:**

This population-based cohort study used administrative data for Ontario, Canada, from April 1, 2002, to December 31, 2019. Included were all 414 337 hospital-based singleton live births and stillbirths occurring between 20 and 42 weeks’ gestation, solely among women residing in an urban neighborhood of the lowest income quintile; all women were receiving universal health care insurance. Statistical analysis was performed from December 2021 to March 2022.

**Exposures:**

Nonrefugee immigrant status vs nonimmigrant status.

**Main Outcomes and Measures:**

The primary outcome, SMM-M, was a composite outcome of potentially life-threatening complications or mortality occurring within 42 days of the index birth hospitalization. A secondary outcome was SMM severity, approximated by the number of SMM indicators (0, 1, 2 or ≥3 indicators). Relative risks (RRs), absolute risk differences (ARDs), and odds ratios (ORs) were adjusted for maternal age and parity.

**Results:**

The cohort included 148 085 births to immigrant women (mean [SD] age at index birth, 30.6 [5.2] years) and 266 252 births to nonimmigrant women (mean [SD] age at index birth, 27.9 [5.9] years). Most immigrant women originated from South Asia (52 447 [35.4%]) and the East Asia and Pacific (35 280 [23.8%]) regions. The most frequent SMM indicators were postpartum hemorrhage with red blood cell transfusion, intensive care unit admission, and puerperal sepsis. The rate of SMM-M was lower among immigrant women (2459 of 148 085 [16.6 per 1000 births]) than nonimmigrant women (4563 of 266 252 [17.1 per 1000 births]), equivalent to an adjusted RR of 0.92 (95% CI, 0.88-0.97) and an adjusted ARD of −1.5 per 1000 births (95% CI, −2.3 to −0.7). Comparing immigrant vs nonimmigrant women, the adjusted OR of having 1 SMM indicator was 0.92 (95% CI, 0.87-0.98), the adjusted OR of having 2 indicators was 0.86 (95% CI, 0.76-0.98), and the adjusted OR of having 3 or more indicators was 1.02 (95% CI, 0.87-1.19).

**Conclusions and Relevance:**

This study suggests that, among universally insured women residing in low-income urban areas, immigrant women have a slightly lower associated risk of SMM-M than their nonimmigrant counterparts. Efforts aimed at improving pregnancy care should focus on all women residing in low-income neighborhoods.

## Key Points

**Question:**

Among women residing exclusively in low-income neighborhoods, does severe maternal morbidity or mortality differ between immigrant vs nonimmigrant women?

**Findings:**

This population-based cohort study comprised 414 337 births in Ontario, Canada, among 312 175 women, all with universal health care insurance, residing in low-income neighborhoods. Immigrants had a slightly lower rate than nonimmigrants of potentially life-threatening complications (severe maternal morbidity) or mortality during birth or the postpartum period (16.6 vs 17.1 per 1000 births).

**Meaning:**

This study suggests that, within low-income neighborhoods, immigrant women may have a slightly lower risk than their nonimmigrant counterparts of severe maternal morbidity or mortality.

## Teaser

This population-based cohort study compares the risk of severe maternal morbidity or mortality between immigrant and nonimmigrant women residing exclusively within low-income neighborhoods in Ontario, Canada.

## Introduction

Maternal morbidity and mortality are important public health indicators of maternal health and quality of health care.^1^ Within industrialized nations, maternal mortality is very rare—approximately 8 deaths per 100 000 live births,^2^ largely due to direct obstetric complications or, indirectly, from a preexisting or new disease exacerbated by pregnancy.^3,4^

Given the infrequency of maternal deaths, research and public health surveillance in high-income countries have focused on a composite of severe maternal morbidity (SMM),^1^ identified by conditions along a continuum to maternal mortality, including life-threatening and disabling diseases, organ dysfunction, and/or receipt of invasive therapy,^2^ most often arising during childbirth and up to 42 days thereafter.^1^ Severe maternal morbidity is a validated proxy of maternal mortality and prolonged hospital length of stay.^5^ Some SMM indicators are prevalent and, at times, preventable.^1,2,6,7^

Although the occurrence of SMM may be increasing,^8,9^ most studies have focused on proximal risk factors (eg, medical conditions, mode of delivery, and hospital-level factors),^10^ which may themselves be influenced by upstream determinants of health, such as the social, economic, and physical characteristics of where a mother lives and works, including neighborhood income level.^11,12,13,14^ Living in a low-income area and migration are each associated with adverse pregnancy outcomes.^11,15,16,17,18^ Residing in an economically deprived area may limit the availability, access, and quality of goods, education, and health and social services^19,20,21^; in addition, immigrants have migration experiences that nonimmigrants do not.^22,23^ Prior studies from the US, UK, Canada, and Australia made comparisons only across area-level income groups, rather than restricting to low-income areas, where the risk of adverse birth outcomes is greater compared with higher-income areas^24,25^ and where SMM may be more pronounced, thereby warranting additional pregnancy care considerations.^9,10,11,13,26,27^

To our knowledge, there are no existing studies comparing the risk of SMM or maternal mortality (SMM-M) among immigrant and nonimmigrant women, specifically looking at variability within low-income neighborhoods.^6,28,29,30^ Ontario provides a unique setting to undertake such a study because it is the most populous and multiethnic province in Canada, receiving 53% of all female immigrants to the nation,^31^ many from source countries where SMM-M is common,^32^ and who often settle in low-income urban areas on arrival.^31^ Moreover, Ontario provides universal health care to all residents, including permanent residents. Consequently, this study compared the risk of SMM-M between immigrant and nonimmigrant women residing exclusively within low-income neighborhoods. Denoting migration status as a single measure (ie, immigrant vs nonimmigrant) can mask birth outcome disparities.^6,33^ Accordingly, we further evaluated SMM-M in association with maternal world region of birth and country of birth, as well as the immigrant woman’s duration of residence in Ontario.

## Methods

### Study Design, Setting, and Participants

This retrospective population-based cohort study was completed in Ontario, Canada, from April 1, 2002, to December 31, 2019. Included were all hospital singleton live births and stillbirths from 20 to 42 weeks’ gestation, among females aged 15 to 50 years at the index birth who had a valid Ontario Health Insurance Plan number and who were currently residing in the lowest-income quintile 1 urban neighborhood. Eligible women with more than 1 singleton birth during the study period were included, and each birth was considered an index birth. Reasons for exclusion are otherwise listed in eTable 1 and eFigure in Supplement 1. Ethics approval was received from the Health Sciences Research Ethics Board at the University of Toronto. This study followed the Strengthening the Reporting of Observational Studies in Epidemiology (STROBE) reporting guideline.

### Data Sources

We used administrative data at ICES in Toronto, Ontario, Canada, an independent, nonprofit research institute whose legal status under Ontario’s health information privacy law allows it to collect and analyze health care and demographic data, without consent, for health system evaluation and improvement. Data sets used for this study are valid and reliable sources for perinatal research and are described in eTable 2 in Supplement 1.^34,35,36,37,38^ These deidentified data sets were linked using unique encoded identifiers and were analyzed at ICES.

All births were identified in the MOMBABY (Linked Delivering Mothers and Newborns) database, which captures hospital labor and delivery records for approximately 98% of Ontario births. Immigrant status was determined using the Immigration, Refugees and Citizenship Canada Permanent Resident database. Neighborhood income quintile was determined using Statistics Canada Postal Code Conversion File Plus (PCCF+, version 7B), based on a woman’s residential postal code at the time of the index birth.^39^

### Study Exposures

The primary exposure compared nonrefugee immigrant women (henceforth, called “immigrant women”) with nonimmigrant women, based on a woman’s birthplace and migration status, prior to the index birth. Refugees or women with other immigrant status, and women concomitantly classified as both a nonimmigrant and immigrant woman, were omitted. Also excluded were immigrants who were missing a permanent residency date (eg, “landing date”), or whose permanent residency date was improbable because it preceded their own date of birth or was after the index delivery date (eTable 1 in Supplement 1).

Secondary exposures included maternal world region of birth, country of birth, and duration of residence in Ontario. Maternal world region of birth was grouped according to a modified version of the United Nations classification scheme: Canada, Caribbean, East Asia and Pacific, Latin America, Middle East and North Africa, South Asia, sub-Saharan Africa, and Western nations and Europe.^40,41^ Specific maternal country of birth was then explored for the 10 countries contributing the greatest number of births in Ontario during the study period. Maternal duration of residence in Ontario, analyzed only among immigrant women, was the number of years between their permanent residency date in Ontario and their index birth hospitalization date, categorized as less than 5 years (reference), 5 to 9 years, 10 to 14 years, or 15 years or longer.^22^

### Outcomes

The primary study outcome was a composite of SMM or all-cause maternal mortality—SMM-M—arising in the index birth hospitalization or up to 42 days thereafter. Severe maternal morbidity, validated by the Canadian Perinatal Surveillance System,^42,43^ was adapted herein to include 40 unique indicators based on the *International Statistical Classification of Diseases and Related Health Problems, Tenth Revision, Canada*, diagnostic codes^44^ and the Canadian Classification of Health Interventions^45^ procedural codes (eg, intensive care unit admission, eclampsia, and use of assisted ventilation) (eTable 1 in Supplement 1).

A secondary outcome was an approximation of SMM severity, marked by the number of SMM indicators, including maternal mortality, present at the index birth and up to 42 days thereafter,^2^ categorized as 0 (reference), 1, 2, or 3 or more indicators.

### Statistical Analysis

Statistical analysis was performed from December 2021 to March 2022. Mean values and proportions were contrasted between immigrant and nonimmigrant women using standard differences, with a value greater than 0.10 considered to be an important difference.^46^ The 20 most common SMM indicators were also shown by immigrant status.

Modified Poisson regression with a robust error variance and generalized estimating equations were used to estimate relative risks (RRs) and absolute risk differences (ARDs) and 95% CIs for the outcome of SMM-M, comparing immigrant vs nonimmigrant women.^47,48,49^ Generalized estimating equations, with an exchangeable correlation structure, accounted for correlation among women who had more than 1 birth during the study period.^50^ A similar modeling approach was used to analyze maternal world region of birth and country of birth, with Canadian-born women serving as the reference. All of these models were adjusted for maternal age (15-19, 20-29, 30-39, or 40-50 years) and parity (0, 1, 2, or ≥3 births) at the index birth.

For the outcome of the number of SMM indicators (0, 1, 2, or ≥3), odds ratios (ORs) were generated using multinomial logistic regression with a robust error variance and generalized estimating equations (independent correlation structure), comparing immigrant and nonimmigrant women. Odds ratios were adjusted for maternal age and parity.

In the immigrant-only analysis of maternal duration of residence in Ontario and the risk of SMM-M, RRs were adjusted for maternal age, parity, and world region of birth, as well as the following variables documented at their arrival date to Canada: era (1985-1990, 1991-2001, 2002-2010, or 2011-2017), highest level of education (secondary school or less, trade certificate with no university or some university, university degree, or graduate degree), Canadian language ability (English and/or French or neither), and immigration class (economic or sponsored family).

Statistical significance was set at a 2-sided *P* < .05. All analyses were performed using SAS, version 9.4 software (SAS Institute Inc).

## Results

Of 2 374 755 overall births in the study period, the derived cohort comprised 414 337 births (148 085 births to immigrant women [mean (SD) age at index birth, 30.6 (5.2) years] and 266 252 births to nonimmigrant women [mean (SD) age at index birth, 27.9 (5.9) years]) (Table 1) among 312 175 women who were residing in a lowest-income quintile 1 urban neighborhood at the time of the index birth (eFigure in Supplement 1).

**Table 1.** Characteristics of Births Among Immigrant and Nonimmigrant Women Residing in a Lowest-Income Quintile Urban Neighborhood

Characteristic	Women, No. (%)^a^		Standardized difference^c^
	Immigrant (n = 148 085)^b^	Nonimmigrant (n = 266 252)	
Mother			
Duration of residence in Canada at the index birth, median (IQR), y	4.9 (2.3-9.9)	NA	NA
Age at the index birth, mean (SD), y	30.6 (5.2)	27.9 (5.9)	0.50
Age category at the index birth, y			
15-19	1678 (1.1)	20 120 (7.6)	0.32
20-29	61 037 (41.2)	142 247 (53.4)	0.25
30-39	78 498 (53.0)	96 997 (36.4)	0.34
40-50	6872 (4.6)	6888 (2.6)	0.11
Parity category			
0	59 409 (40.1)	121 509 (45.6)	0.11
1	55 497 (37.5)	85 410 (32.1)	0.11
2	21 885 (14.8)	36 389 (13.7)	0.03
≥3	11 294 (7.6)	22 944 (8.6)	0.04
Newborn			
Birth type			
Live birth	148 050 (99.98)	266 191 (99.98)	0.00
Stillbirth	35 (0.02)	61 (0.02)	0.00
Birth weight, mean (SD), g	3254.1 (544.2)	3351.1 (583.1)	0.17
Gestational age, mean (SD), wk	38.7 (1.9)	38.8 (2.0)	0.03
Preterm birth <37 wk	9465 (6.4)	20 035 (7.5)	0.05

Abbreviation: NA, not applicable.

^a^ Data are limited to those who had a singleton hospital live birth or stillbirth at 20 weeks and 0 days’ to 42 weeks and 0 days’ gestation in Ontario, Canada, 2002 to 2019.

^b^ Excludes refugee immigrants.

^c^ A standardized difference of greater than 0.10 is considered to be clinically meaningful.

Nonimmigrant women were more likely to be nulliparous, and their infants, on average, had a higher birth weight (Table 1). Most immigrant women originated from South Asia (52 447 [35.4%]) and the East Asia and Pacific region (35 280 [23.8%]), and 111 651 immigrants (75.4%) had resided in Ontario for less than 10 years (eTable 3 in Supplement 1). Additional characteristics of immigrant women are presented in eTable 3 in Supplement 1.

Severe maternal morbidity or mortality occurred among 2459 of 148 085 immigrant women (16.6 per 1000 births) vs 4563 of 266 252 nonimmigrant women (17.1 per 1000 births), equivalent to an unadjusted RR of 0.97 (95% CI, 0.92-1.02), an adjusted RR (aRR) of 0.92 (95% CI, 0.88-0.97), and an adjusted ARD of −1.5 cases per 1000 births (95% CI, −2.3 to −0.7). The ranking of the 20 most frequent SMM indicators was largely similar among immigrant and nonimmigrant women, dominated by severe postpartum hemorrhage, intensive care unit admission, and puerperal sepsis (eTable 4 in Supplement 1).

Immigrant women also had a lower adjusted OR than their nonimmigrant counterparts for experiencing 1 SMM indicator (0.92 [95% CI, 0.87-0.98]) or 2 SMM indicators (0.86 [95% CI, 0.76-0.98]), but not 3 or more SMM indicators (1.02 [95% CI, 0.87-1.19]) (Table 2).

**Table 2.** Odds Ratios for Having a Higher Number of SMM Indicators in the Index Birth Hospitalization or Up to 42 Days Thereafter, by Comparing Immigrant vs Nonimmigrant Women Residing in a Lowest-Income Quintile Urban Neighborhood

Exposure group	No.^c^	Outcome of 1 SMM indicator^a^^,^^b^			Outcome of 2 SMM indicators^a^^,^^b^			Outcome of ≥3 SMM indicators^a^^,^^b^		
		No. (per 1000 births)	OR (95% CI)		No. (per 1000 births)	OR (95% CI)		No. (per 1000 births)	OR (95% CI)	
			Unadjusted^d^	Adjusted^d^^,^^e^		Unadjusted^d^	Adjusted^d^^,^^e^		Unadjusted^d^	Adjusted^d^^,^^e^
Nonimmigrants	266 252	3464 (13.0)	1 [Reference]	1 [Reference]	706 (2.7)	1 [Reference]	1 [Reference]	393 (1.5)	1 [Reference]	1 [Reference]
Immigrants^f^	148 085	1842 (12.4)	0.96 (0.90-1.01)	0.92 (0.87-0.98)	369 (2.5)	0.94 (0.83-1.07)	0.86 (0.76-0.98)	248 (1.7)	1.13 (0.97-1.33)	1.02 (0.87-1.19)

Abbreviations: OR, odds ratio; SMM, severe maternal morbidity.

^a^ A total of 261 689 nonimmigrant women (98.3%) had 0 SMM indicators.

^b^ A total of 145 626 immigrant women (98.3%) had 0 SMM indicators.

^c^ Data are limited to those who had a singleton hospital live birth or stillbirth at 20 weeks and 0 days’ to 42 weeks and 0 days’ gestation in Ontario, Canada, 2002 to 2019.

^d^ Using multinomial logistic regression. Generalized estimating equations with an independent correlation structure accounted for correlated errors due to potentially more than 1 birth clustered within the same mother.

^e^ Adjusted for maternal age (15-19, 20-29, 30-39, or 40-50 years), and parity (0, 1, 2, or ≥3 births).

^f^ Excludes refugee immigrants.

When divided by maternal world region of birth, relative to Canadian-born women, the aRRs for SMM-M were lower among immigrant women from Western nations and Europe (0.73 [95% CI, 0.63-0.85]), South Asia (0.84 [95% CI, 0.78-0.91]), and East Asia and the Pacific (0.83 [95% CI, 0.76-0.91]) (Table 3). Conversely, the aRRs were higher among women originating from the Caribbean (1.35 [95% CI, 1.20-1.53]) and sub-Saharan Africa (1.38 [95% CI, 1.22-1.56]). Among the top 10 countries of origin, women from Bangladesh (1.43 [95% CI, 1.20-1.70]), Jamaica (1.50 [95% CI, 1.30-1.73]), and Ghana (1.67 [95% CI, 1.34-2.08]) had the highest aRRs for SMM-M compared with Canadian-born women, while those from Sri Lanka, China, and India were at significantly lower risk (eTable 5 in Supplement 1).

**Table 3.** Risk of Severe Maternal Morbidity or Maternal Mortality Arising in the Index Birth Hospitalization or Up to 42 Days Thereafter by World Region of Origin

Maternal world region of origin exposure group	No.^a^^,^^b^	No. (per 1000 births)	Relative risk (95% CI)		Adjusted absolute risk difference, per 1000 births (95% CI)^c^^,^^d^
			Unadjusted^c^	Adjusted^c^^,^^d^	
Canadian born	266 252	4563 (17.1)	1 [Reference]	1 [Reference]	0 [Reference]
Caribbean	12 032	281 (23.4)	1.37 (1.21 to 1.54)	1.35 (1.20 to 1.53)	5.9 (3.1 to 8.6)
East Asia and Pacific	35 280	584 (16.6)	0.97 (0.89 to 1.05)	0.83 (0.76 to 0.91)	−3.1 (−4.6 to −1.7)
Latin America	9685	156 (16.1)	0.94 (0.80 to 1.10)	0.91 (0.77 to 1.06)	−1.5 (−4.0 to 1.0)
Middle East and North Africa	13 812	213 (15.4)	0.90 (0.78 to 1.03)	0.87 (0.76 to 1.00)	−2.1 (−4.1 to 0.0)
South Asia	52 447	759 (14.5)	0.84 (0.78 to 0.91)	0.84 (0.78 to 0.91)	−2.8 (−3.9 to −1.7)
Sub-Saharan Africa	11 795	288 (24.4)	1.43 (1.27 to 1.61)	1.38 (1.22 to 1.56)	6.1 (3.3 to 8.8)
Western nations and Europe	12 996	177 (13.6)	0.79 (0.68 to 0.92)	0.73 (0.63 to 0.85)	−4.9 (−6.9 to −3.0)

^a^ Excludes 38 women missing world region of birth.

^b^ Data are limited to women residing in the lowest income quintile urban neighborhood, and who had a singleton hospital live birth or stillbirth at 20 weeks and 0 days’ to 42 weeks and 0 days’ gestation in Ontario, Canada, 2002 to 2019.

^c^ Using modified Poisson regression with a robust error variance. Generalized estimating equations with an exchangeable correlation structure accounted for correlated errors due to potentially more than 1 birth clustered within the same mother.

^d^ Adjusted for maternal age (15-19, 20-29, 30-39, or 40-50 years) and parity (0, 1, 2, or ≥3 births).

When restricting analysis to immigrant women, the rate of SMM-M was lower among those residing in Ontario less than 5 years (16.3 per 1000), 5 to 9 years (15.6 per 1000), and 10 to 14 years (16.9 per 1000) compared with those who had arrived at least 15 years earlier (19.7 per 1000) (Table 4). The aRR for SMM-M did not differ among immigrant women residing in Ontario for 5 to 9 years vs less than 5 years, while the aRR increased with duration of residence, from 1.18 (95% CI, 1.02-1.37) for 10 to 14 years to 1.31 (95% CI, 1.10-1.57) for 15 or more years—each compared with those living in Ontario for less than 5 years (Table 4).

**Table 4.** Risk of Severe Maternal Morbidity or Maternal Mortality Arising in the Index Birth Hospitalization or Up to 42 Days Thereafter, Comparing Immigrant Women by Duration of Residence in Ontario

Duration of residence in Ontario among immigrant women	No. (%)^a^^,^^b^	No. (per 1000 births)	Relative risk (95% CI)		Adjusted absolute risk difference, per 1000 births (95% CI)^c^^,^^d^
			Unadjusted^c^	Adjusted^c^^,^^d^	
<5 y	74 343 (50.4)	1213 (16.3)	1 [Reference]	1 [Reference]	0 [Reference]
5-9 y	36 828 (25.0)	576 (15.6)	0.96 (0.87 to 1.06)	1.08 (0.97 to 1.20)	1.2 (−0.6 to 3.0)
10-14 y	18 953 (12.9)	320 (16.9)	1.04 (0.92 to 1.17)	1.18 (1.02 to 1.37)	2.7 (0.1 to 5.4)
≥15 y	17 400 (11.8)	343 (19.7)	1.21 (1.07 to 1.36)	1.31 (1.10 to 1.57)	4.8 (1.4 to 8.2)

^a^ Excludes 266 252 nonimmigrant women and 466 immigrant women missing highest level of education at arrival to Canada, 39 immigrant women missing highest level of education and Canadian language ability at arrival to Canada, 38 women missing world region of birth at arrival to Canada, and 18 immigrant women missing Canadian language ability at arrival to Canada.

^b^ Data are restricted to immigrant women residing in the lowest income quintile urban neighborhood, and who had a singleton hospital live birth or stillbirth at 20 weeks and 0 days’ to 42 weeks and 0 days’ gestation in Ontario, Canada, 2002 to 2019.

^c^ Using modified Poisson regression with a robust error variance. Generalized estimating equations with an exchangeable correlation structure accounted for correlated errors due to potentially more than 1 birth clustered within the same mother.

^d^ Adjusted for maternal age (15-19, 20-29, 30-39, or 40-50 years), parity (0, 1, 2, or ≥3 births), world region of birth (Caribbean, East Asia and Pacific, Latin America, Middle East and North Africa, South Asia, sub-Saharan Africa, or Western nations and Europe), and the following variables at arrival to Canada: highest level of education (secondary school or less, trade certificate with no university or some university, university degree, or graduate degree), Canadian language ability (English and/or French or neither), era (1985-1990, 1991-2001, 2002-2010, or 2011-2017), and immigration class (economic or sponsored family).

## Discussion

In this large population-based study of immigrant and Canadian-born women residing in low-income urban neighborhoods, immigrant women had a slightly lower associated risk than nonimmigrant women of SMM-M. However, this association was due to the lower risk of SMM-M among women originating from Western nations and Europe, South Asia, and the East Asia and Pacific regions, as well as more recent immigrants. In contrast, the risk of SMM-M was much higher for immigrant women from the Caribbean and sub-Saharan Africa than their Canadian-born counterparts.

### Other Studies

This study is novel in its exclusive focus on quantifying the risk of SMM-M among women residing in low-income urban neighborhoods. Previous studies did investigate the risk of SMM by migrant status or race and ethnicity, but most did not examine area-level income measures.^11,13,51,52^ One prior study observed a progressively lower risk of maternal placental syndromes among immigrant woman with shorter duration of residence in the host country preceding the pregnancy, including in the lowest and highest income areas, which is consistent with our findings.^17^ However, other research on migration and the risk of SMM—irrespective of area-level income—has yielded differing findings. For example, 2 Canadian studies reported no difference in the risk of SMM by immigrant status, although the rates of SMM in those studies were lower than those observed herein among women residing exclusively in low-income areas.^6,29^ Studies from the Netherlands, Sweden, and the US found a higher risk of SMM among immigrant women compared with native-born women.^11,30,53,54^ Our findings are in keeping with those of others, which showed that the risk of maternal morbidity tends to vary by maternal world region and country of birth. Specifically, other studies also noted a higher risk among immigrant women from sub-Saharan Africa and the Caribbean than among immigrant women from industrialized and Western nations,^18,53,54^ or compared with nonimmigrant women.^6,28^ Beyond having restricted our sample to women residing in low-income urban areas, our findings may differ from prior research based on the method used to define migrant subgroups or SMM-M, choice of the reference group, the destination country,^33^ and the availability of universal primary and prenatal care.^28,55^

The present study introduces the approach of estimating the risk of important health outcomes—in this case, SMM-M—within area-level constrained income groups. Doing so may help clarify the association between migration and SMM-M, and, especially, the identification of those who are at highest risk. Overall, these findings suggest that immigrant women residing in low-income neighborhoods may fare better than expected for severe pregnancy outcomes compared with their nonimmigrant counterparts. Three epidemiologic phenomena may partly explain this finding. The first is the healthy immigrant effect, in which recent or first-generation immigrant individuals are healthier than native-born individuals in the host country. However this initial health advantage for immigrant individuals disappears with increased length of residency.^56,57^ This may be attributed to selective migration, in which healthier or more resilient people tend to migrate, and/or that immigration policies in the host country select migrants with such attributes.^57^ Immigrant groups may bring cultural norms and values that initially promote healthier lifestyles and behaviors, with strong family connections that buffer the effects of stress.^58^ Relatedly, the immigrant paradox posits that some migrant groups experience substantially better health than the average citizen within the host country, despite having lower income and greater barriers to health care access.^57,59,60^ A second explanation is that migrant groups have better health states because their socioeconomic position—marked by greater net income, educational level, and health literacy—is actually higher than what their overall neighborhood income measure reflects. Third, the convergence hypothesis suggests that, with longer duration of residence, most immigrants will adopt an unhealthy lifestyle that becomes indistinguishable from that of the majority population in the host country.^57^ Likewise, our study suggests that increasing duration of residence within an economically disadvantaged area is associated with a progressively higher risk of SMM-M among immigrant women.

Immigrant women have diverse experiences that may be differentially associated with their health states before and during pregnancy, such as migration factors and barriers to accessing pregnancy care services.^60,61,62^ This is underscored by the current observation that the risk of SMM-M differs by maternal birthplace. This further suggests that the healthy immigrant effect is not experienced by all immigrant groups. Whether this difference is associated with social, cultural, or economic factors from the place of origin; cultural factors of the destination country; and/or time spent in the destination country requires clarification.^6^

### Public Health and Clinical Relevance

These findings highlight the importance of designing prepregnancy, antenatal, and peripartum care initiatives that are inclusive of immigrant and nonimmigrant women alike who are residing in low-income neighborhoods. To achieve this goal, more details are required not only about the needs of various immigrant groups but also those who have lived in a low-income area for more than 1 generation. Beyond a woman’s race or ethnicity, there is also a need to consider her cultural practices and health literacy, as well as individual-level income, type of employment, and social mobility (to determine if she has recently lost or gained wealth). Because Ontario has universal health care, immigrant and nonimmigrant women should, theoretically, have equal access to health care; however, this needs verification, alongside a better understanding of the barriers and facilitators to health care delivery, such as travel distance to appointments, child care, partner and extended family supports, language facility, and timing of initiation of antenatal care. Future studies should also investigate the potential of community-based models for antenatal and peripartum care intended to improve pregnancy outcomes among all women residing in low-income neighborhoods because this approach has yielded positive outcomes in the UK.^63,64^

### Limitations

This study has several limitations. Since only singleton births were included in the cohort, our findings may not be applicable to women with multifetal pregnancies because their maternal and perinatal risk factors and birth outcomes can differ from those of singleton pregnancies.^65^ An immigrant woman not linked to the Immigration, Refugees and Citizenship Canada Permanent Residents database (ie, who had no record of immigration) was classified as a nonimmigrant (ie, Canadian born), potentially leading to a small proportion of immigrant women being misclassified as nonimmigrant women. Such misclassification would be expected to bring the hypothesis toward the null. Refugees or immigrants with other special status were not included in this study because they may have lacked an Ontario Health Insurance Plan number necessary for data linkage. Given that refugees often have different migration experiences (eg, nonvoluntary migration and secondary migration) than nonrefugee immigrant groups, the current findings may not be generalizable to the former subgroup of immigrants.

A neighborhood income quintile reflects area- and individual-level income status.^66^ Data about individual-level income were unavailable herein, however. A woman lacking an up-to-date residential address may have been incorrectly assigned to the wrong neighborhood income quintile. Details about some sociodemographic factors identified among immigrant women at their arrival (eg, educational level and language ability) were not available for nonimmigrant women, while early-life exposures and the obstetrical events before migration were unknown among immigrant women. Psychosocial factors, employment conditions, behavioral factors (eg, smoking or substance use), measures of obesity, and of quality of care were unknown for all women in the study. Because SMM-M is a composite measure, certain immigrant and nonimmigrant women may be differentially more prone to certain conditions. Finally, the present study focused on women living in urban areas. How immigrant and nonimmigrant women in rural areas compare requires ongoing study because there exists a divide in health care access and health measures between urban and rural residents,^67^ including those residing in low-income rural areas.^68^

## Conclusions

The results of this study suggest that, among women residing in low-income urban neighborhoods in Ontario, Canada, who have universal health care insurance, immigrant women have a slightly lower overall risk of SMM-M than nonimmigrant women. Even so, there is heterogeneity in the risk of SMM-M between various immigrant source groups. Initiatives aimed at improving pregnancy care should focus on immigrant and nonimmigrant women residing in low-income urban areas.

## Notes

Supplement 1. **eTable 1. **Cohort Entry and Exclusion Criteria, Methods and Coding to Identify Study Outcomes **eTable 2. **List of ICES Databases Used in the Current Study **eFigure. **Flow Diagram of Cohort Creation **eTable 3. **Characteristics of the 148,085 Nonrefugee Immigrant Women at Their Arrival to Canada, and Their Duration of Residence in Ontario **eTable 4. **Risk of Severe Maternal Morbidity (SMM) or Maternal Mortality Arising in the Index Delivery Hospitalization or up to 42 Days Thereafter, and the Top-20 SMM Indicators **eTable 5. **Risk of Severe Maternal Morbidity or Maternal Mortality Arising in the Index Delivery Hospitalization or up to 42 Days Thereafter, Comparing Immigrants From 10 Countries With the Greatest Number of Births in Ontario to Canadian-Born Women

Supplement 2.
Data Sharing Statement
