# Supplementary material for: Severe Maternal Morbidity and Mortality Among Immigrant and Canadian-Born Women Residing Within Low-Income Neighborhoods in Ontario, Canada
Source: JAMA Netw Open. 2023 Feb 16;6(2):e2256203. doi: 10.1001/jamanetworkopen.2022.56203 (PMC9936351; doi:10.1001/jamanetworkopen.2022.56203)
Supplement: Supplement 1. — eTable 1. Cohort Entry and Exclusion Criteria, Methods and Coding to Identify Study Outcomes eTable 2. List of ICES Databases Used in the Current Study eFigure. Flow Diagram of Cohort Creation eTable 3. Characteristics of the 148,085 Nonrefugee Immigrant Women at Their Arrival to Canada, and Their Duration of Residence in Ontario eTable 4. Risk of Severe Maternal Morbidity (SMM) or Maternal Mortality Arising in the Index Delivery Hospitalization or up to 42 Days Thereafter, and the Top-20 SMM Indicators eTable 5. Risk of Severe Maternal Morbidity or Maternal Mortality Arising in the Index Delivery Hospitalization or up to 42 Days Thereafter, Comparing Immigrants From 10 Countries With the Greatest Number of Births in Ontario to Canadian-Born Women [file jamanetwopen-e2256203-s001.pdf]

Supplementary Online Content

Jairam JA, Vigod SN, Siddiqi A, et al. Severe maternal morbidity and mortality among immigrant and Canadian-born women residing within low-income neighborhoods in Ontario, Canada. *JAMA Netw Open.* 2023;6(2):e2256203. doi:10.1001/jamanetworkopen.2022.56203

eTable 1. Cohort Entry and Exclusion Criteria, Methods and Coding to Identify Study Outcomes
eTable 2. List of ICES Databases Used in the Current Study
eFigure. Flow Diagram of Cohort Creation
eTable 3. Characteristics of the 148,085 Nonrefugee Immigrant Women at Their Arrival to Canada, and Their Duration of Residence in Ontario
eTable 4. Risk of Severe Maternal Morbidity (SMM) or Maternal Mortality Arising in the Index Delivery Hospitalization or up to 42 Days Thereafter, and the Top-20 SMM Indicators
eTable 5. Risk of Severe Maternal Morbidity or Maternal Mortality Arising in the Index Delivery Hospitalization or up to 42 Days Thereafter, Comparing Immigrants From 10 Countries With the Greatest Number of Births in Ontario to Canadian-Born Women

This supplementary material has been provided by the authors to give readers additional information about their work.

© 2023 Jairam JA et al. *JAMA Network Open.*

eTable 1. Cohort Entry and Exclusion Criteria, Methods and Coding to Identify Study Outcomes

Assessment	Timing	Disease, procedure or condition	ICD-10-CA or CCI codes in CIHI-DAD	Other sources
Inclusion criteria	April 1, 2002 to December 31, 2019, at the time of the mother's index delivery hospitalization date	● All hospital-based singleton livebirths or stillbirths at $20^{0/7}$ to $42^{0/7}$ weeks' gestation, in Ontario, Canada, among non-refugee immigrant & non-immigrant women living in a low-income urban neighbourhood in Ontario (income quintile 1 [Q1]).<br>● Women aged 15 to 50 years, with a valid OHIP number/IKN from MOMBABY.<br>● Stillbirth: newborn with no signs of life at birth or *in utero*, and ≥ $20^{0/7}$ weeks' gestation and ≥ 250 g at birth.	MOMBABY (links the hospital admission records of delivering mothers and newborns in CIHI-DAD)	IRCC-PRD, RPDB
	At the time of index delivery hospitalization	Mother's postal code was used to derive neighbourhood income Q.	--	RPDB, PCCF+, Statistic Canada census data
Exclusion criteria	Relative risk time of the index (infant) birth admission	Infant birthweight < 250g or missing	MOMBABY:<br>a. B_WEIGHT< 250<br>b. B_WEIGHT not in MOMBABY	
	Same	Infant gestational age at birth missing	MOMBABY:<br>a. B_GESTWKS_DEL not in MOMBABY<br>b. gestational age <20 or ≥43 weeks	--
	At the time of index delivery hospitalization	Women <15 or >50 years or age missing	--	RPDB: M_IKN age < 15 or > 50 or missing
	Same	Women who were a non-Ontario resident	--	RPDB: M_IKN non-Ontario resident (substr (prcddablk,1,2 ne '35')
	Same	Women who had an invalid OHIP number or hospital number & infants who had an invalid OHIP number	MOMBABY/RPDB: Invalid M_IKN<br>a. VALIKN ne 'V'<br>b. M_IKN not in RPDB (according to no sex & no bdate)<br>c. M_IKN with sex ='M' in RPDB	--
	Same	Women ineligible for OHIP	--	RPDB
	Same	Women from Q2, Q3, Q4, Q5 neighbourhood income Q or missing	--	RPDB, PCCF+, Statistics Canada census data
	Same	Records with warning for mother's or infants' IKN or KEY	MOMBABY/RPBD: warning for IKN/KEY (WARN not ="N"(No Warning)). Include N=no warning	--
	Same	Women living in a rural area	--	RPDB: (rural='0')
	At the time of the index (infant) birth admission	Multiple births	MOMBABY: (M_MULTIBIRTH='T' or B_MULTIBIRTH='T')	--

© 2023 Jairam JA et al. *JAMA Network Open.*

Assessment	Timing	Disease, procedure or condition	ICD-10-CA or CCI codes in CIHI-DAD	Other sources
	Same	Removed successive births if a woman had >1 pregnancy during the study, & current infant birth date was < 161 days apart from the prior infant's birth date	MOMBABY	--
	At the time of arrival to Canada	Refugees, other immigrants, & women classified as non-immigrant & immigrant	--	IRCC-PRD
	Same	Non-refugee immigrants with a landing date prior to their birth date, or after their index delivery hospitalization date, or missing	--	IRCC-PRD, RPDB
Main exposure	At the time of arrival to Canada	Immigrant status: non-refugee immigrant & non-immigrant	--	IRCC-PRD
Secondary exposure	Same	Maternal world region of birth: Canada, Western Nations & Europe, South Asia, Middle East & North Africa, Latin America, East Asia & Pacific, Caribbean, Sub-Saharan Africa	--	IRCC-PRD
	At the time of arrival to Canada & births between 2002-2019	Top 10 maternal countries of birth contributing the greatest number of births in Ontario: Canada, Bangladesh, China, Ghana, Guyana, India, Jamaica, Pakistan, Philippines, Sri Lanka, Vietnam	--	IRCC-PRD
	Mother's landing date in Canada & date of index (infant) birth admission	Duration of residence in ON: subtracted landing date from mother's index delivery hospitalization date divided by 365.25	MOMBABY	IRCC-PRD

© 2023 Jairam JA et al. *JAMA Network Open.*

Assessment	Timing	Disease, procedure or condition	ICD-10-CA or CCI codes in CIHI-DAD	Other sources
Main outcome	At the time of the index delivery hospitalization	Severe maternal morbidity (SMM)	Severe preeclampsia and (HELLP) syndrome:<br>O14.1, or O14.2<br><br>Eclampsia:<br>O15<br><br>Cerebral venous thrombosis in pregnancy, or in the puerperium:<br>O22.5, or O87.3<br><br>Acute fatty liver with red blood cell (RBC) transfusion or plasma transfusion:<br>O26.6 + (CIHI BTREDBC = 1 or CIHI BTPLASMA = 1)<br><br>Pulmonary, cardiac, and CNS complications of anesthesia during pregnancy, the puerperium or labour and delivery:<br>O29.0, O29.1, O29.2, O89.0, O89.1, O89.2, O74.0, O74.1, O74.2 or O74.3	--

© 2023 Jairam JA et al. *JAMA Network Open.*

Assessment	Timing	Disease, procedure or condition	ICD-10-CA or CCI codes in CIHI-DAD	Other sources
			Placenta previa with hemorrhage with RBC transfusion:<br>O44.1 + CIHI BTREDBC = 1<br><br>Placental abruption with coagulation defect:<br>O45.0<br><br>Antepartum hemorrhage with coagulation defect:<br>O46.0<br><br>Intrapartum hemorrhage with coagulation defect:<br>O67.0<br><br>Intrapartum hemorrhage with RBC transfusion:<br>O67 + CIHI BTREDBC = 1<br><br>Rupture of the uterus with RBC transfusion, procedures to the uterus or hysterectomy:<br>(O71.0 or O71.1) + any of the following:<br>• CIHI BTREDBC = 1, or<br>• (1.RM.13, 1.KT.51, 5.PC.91.LA or 5.PC.91.HV) + CIHI BTREDBC = 1, or<br>• (5.MD.60.RC, 5.MD.60.RD, 5.MD.60.KE, 5.MD.60.CB or 1.RM.89.LA[a]), or<br>• 1.RM.87.LA-GX<br><br>[a]**NOTE**: **1.RM.89.LA** is included only if codes 1.PL.74, 1.RS.74 or 1.RS.80 are NOT also present	

© 2023 Jairam JA et al. *JAMA Network Open.*

			Postpartum hemorrhage with RBC transfusion, procedures to the uterus or hysterectomy:<br>O72 + any of the following:<br>• BTREDBC = 1, or<br>• (1.RM.13, 1.KT.51, 5.PC.91.LA or 5.PC.91.HV) + BTREDBC = 1, or<br>• (5.MD.60.RC, 5.MD.60.RD, 5.MD.60.KE, 5.MD.60.CB or 1.RM.89.LA[b]), or<br>• 1.RM.87.LA-GX<br><br>[b]**NOTE**: **1.RM.89.LA** is included only if codes 1.PL.74, 1.RS.74 or 1.RS.80 are NOT also present<br><br>Cardiac conditions:<br>O74.2, O89.1, O90.3, I21, I22, I42, I43, I46, I49.0, I50, J81, 1.HZ.09 or 1.HZ.30<br><br>Obstetric shock:<br>O75.1, R57, T80.5 or T88.6<br><br>Septicemia during labour:<br>O75.3<br><br>Complications of obstetric surgery and procedures:<br>O75.4<br><br>Puerperal sepsis:<br>O85<br><br>Obstetric embolism:<br>O88<br><br>Acute renal failure:<br>O90.4, N17, N19 or N99.0<br><br>Disseminated intravascular coagulation:	

© 2023 Jairam JA et al. *JAMA Network Open.*

Assessment	Timing	Disease, procedure or condition	ICD-10-CA or CCI codes in CIHI-DAD	Other sources
			D65<br><br>Sickle cell anemia with crisis:<br>D57.0	
			Acute psychosis:<br>F53.1 or F23<br><br>Status epilepticus:<br>G41<br><br>Cerebral edema or coma:<br>G93.6 or R40.2<br><br>Cerebrovascular diseases: subarachnoid and intracranial hemorrhage, cerebral infarction, stroke:<br>I60, I61, I62, I63 or I64<br><br>Status asthmaticus:<br>J45.01, J45.11, J45.81 or J45.91<br><br>Adult respiratory distress syndrome:<br>J80	--

© 2023 Jairam JA et al. *JAMA Network Open.*

			<u>Acute abdomen:</u><br>K35, K37, K65, N73.3 or N73.5<br><br><u>Hepatic failure:</u><br>K71 or K72<br><br><u>Assisted ventilation through endotracheal tube:</u><br>1.GZ.31.CA-ND<br><br><u>Assisted ventilation through tracheostomy:</u><br>1.GZ.31.CR-ND<br><br><u>Hysterectomy</u>:<br>5.MD.60.RC, 5.MD.60.RD, 5.MD.60.KE, 5.MD.60.CB, 1.RM.89.LA (exclude if 1.PL.74, 1.RS.74 or 1.RS.80 code also present), 1.RM.87.LA-GX<br><br><u>Dialysis</u>:<br>1.PZ.21<br><br><u>Evacuation of incisional hematoma with RBC transfusion:</u><br>5.PC.73.JS + CIHI BTREDBC = 1<br><br><u>Repair of bladder, urethra, or intestine:</u><br>5.PC.80.JR, 1.NK.80, 1.NM.80<br><br><u>Procedures to the uterus/pelvic vessels with RBC transfusion:</u><br>(1.RM.13, 1.KT.51, 5.PC.91.LA, 5.PC.91.HV) + CIHI BTREDBC = 1<br><br><u>Surgical or manual correction of inverted uterus for vaginal births only:</u><br>5.PC.91.HQ or 5.PC.91.HP, restricted to vaginal births (i.e., absence of caesarean 5.MD.60)	--

© 2023 Jairam JA et al. *JAMA Network Open.*

Assessment	Timing	Disease, procedure or condition	ICD-10-CA or CCI codes in CIHI-DAD	Other sources
			Reclosure of caesarean wound with RBC transfusion:<br>(5.PC.80.JM, 5.PC.80.JH) + CIHI BTREDBC = 1	
			Curettage with RBC transfusion:<br>(5.PC.91.GA, 5.PC.91.GC, 5.PC.91.GD) + CIHI BTREDBC = 1<br><br>Maternal ICU admission:<br>SCU in (‘10’, ’20’, ’25’, ’30’, ’35’, ’40’,’45’,’60’,’80’)	--
	Between 0 & 42 days after the index delivery hospitalization	SMM or all-cause maternal mortality	See above for SMM indicators, and O96 or other all-cause mortality (CIHI-DAD)	RPDB, ORG-D
Secondary outcome	Same	SMM severity: the number of SMM indicators, including all-cause mortality	See above for SMM indicators and O96 or other all-cause mortality (CIHI-DAD)	RPDB, ORG-D
Covariates<br>*Immigrant-only analysis*	At the time of the index delivery hospitalization	Maternal age (years)	--	RPDB
	Same	Parity	MOMBABY	--
	At the time of arrival to Canada	Highest level of education	--	IRCC-PRD
	Same	Canadian language ability	--	IRCC-PRD
	Same	Immigrant class	--	IRCC-PRD
	Same	Year	--	IRCC-PRD
	Same	Age (years): subtracted mother's landing date in Canada from mother's birth date	--	IRCC-PRD
	Same	World region of birth (see above)	--	IRCC-PRD

Abbreviations: CIHI: Canadian Institute for Health Information; DAD: CCI: Canadian Classification of Health Interventions; Discharge Abstract Database; ICD-10-CA: International Classification of Diseases, 10th Revision, Canada; IRCC-PRD: Immigration, Refugees, and Citizenship Canada Permanent Resident Database; OHIP: Ontario Health Insurance Plan; ORG-D: Office of the Registrar General Deaths; PCCF+: Postal code conversion file plus (Statistics Canada); RPDB: Registered Persons Database

© 2023 Jairam JA et al. *JAMA Network Open.*

eTable 2. List of ICES Databases Used in the Current Study

**Dataset name**	**Description**
Canadian Institute for Health Information Discharge Abstract Database (CIHI-DAD)	Captures all in-patient hospital admission records including obstetric deliveries and deaths. Diagnostic codes are based on the *International Statistical Classification of Diseases and Related Health Problems, Tenth Revision, Canada* (*ICD-10-CA*), and procedural codes are based on the *Canadian Classification of Health Interventions (CCI)*.
Linked Delivering Mothers and Newborns (MOMBABY)	Derived from CIHI-DAD, provides linked inpatient hospital admission records of mothers and their infants.
Immigration, Refugees and Citizenship Canada Permanent Residents database (IRCC-PR)	Contains demographic information on all international migrants who obtained permanent residency in Canada from January 1985 to May 2017.
Registered Persons Database (RPDB)	Includes vital status and sociodemographic information about all individuals who have ever received an Ontario Health Insurance Plan (OHIP) number (e.g., date of birth, sex, and postal code).
Postal Code Conversion File Plus (PCCF+)	A digital file that links the Canada Post Corporation (CPC) six-character postal code and Statistics Canada's standard geographic areas (e.g., dissemination area). Area-level income quintiles ranges from Q1 (lowest) to Q5 (highest) income neighbourhoods.
Statistic Canada Census	Information from the Canadian Census, statistical information about the population including population counts and various levels of geography (e.g., census metropolitan areas, communities, census tracts etc.)
Office of the Registrar General - Deaths (ORG-D)	An annual dataset including the date and cause of death, for all deaths registered in Ontario (1990-2021).

© 2023 Jairam JA et al. *JAMA Network Open.*

eFigure. Flow Diagram of Cohort Creation

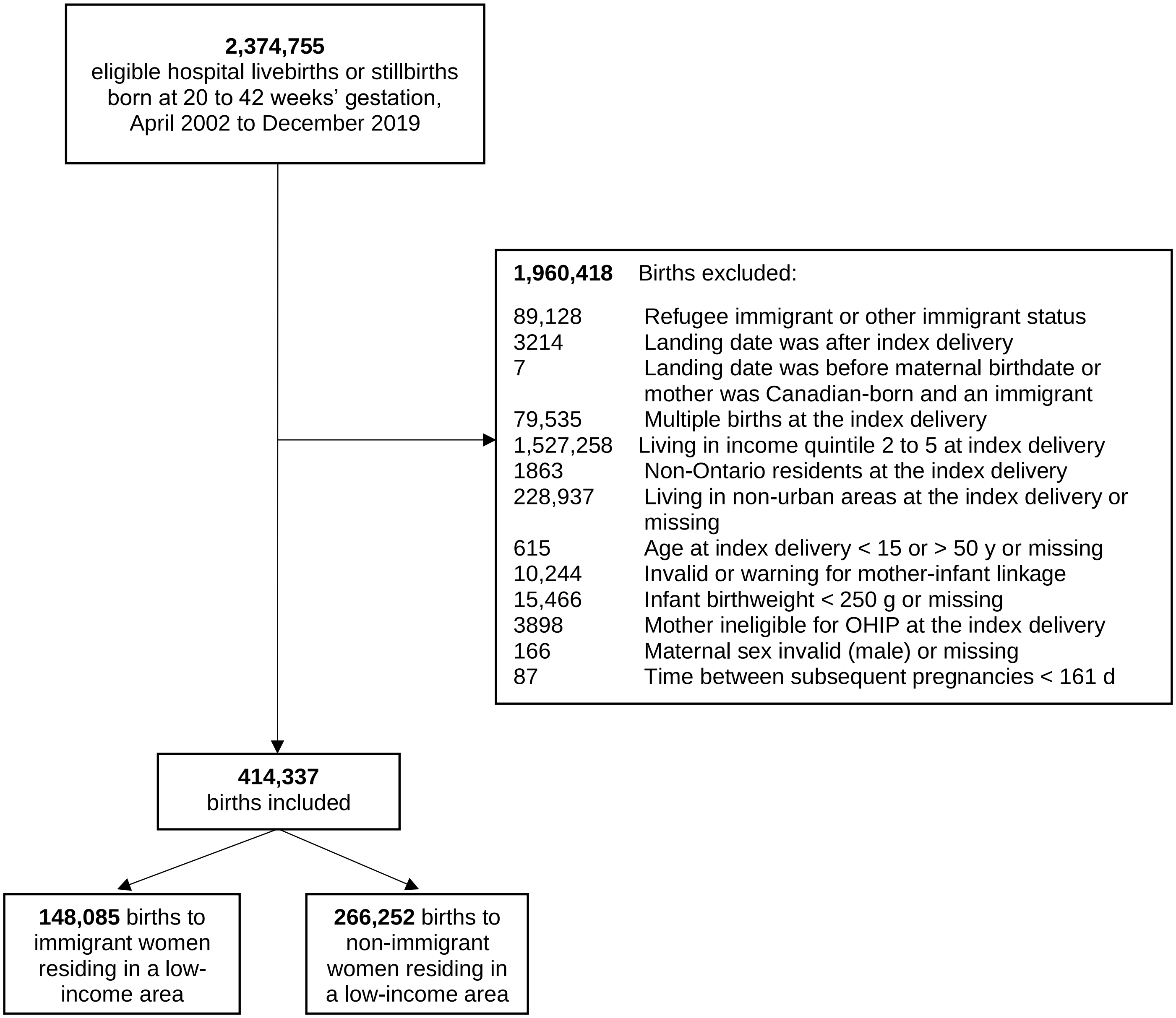

© 2023 Jairam JA et al. *JAMA Network Open.*

eTable 3. Characteristics of the 148,085 Nonrefugee Immigrant Women at Their Arrival to Canada, and Their Duration of Residence in Ontario. Data are limited to women residing in the lowest income quintile urban neighbourhood, and who had a singleton hospital livebirth or stillbirth at 20 to 42 weeks' gestation in Ontario, Canada, 2002 to 2019. All data are presented as a number (%) unless otherwise indicated.

**Characteristic**	
World region of origin	
Caribbean	12,032 (8.1)
East Asia & Pacific	35,280 (23.8)
Western Nations & Europe	12,996 (8.8)
Latin America	9685 (6.5)
Middle East & North Africa	13,812 (9.3)
South Asia	52,447 (35.4)
Sub-Saharan Africa	11,795 (8.0)
Missing	38 (0.03)
Year of arrival to Canada	
1985-1990	6592 (4.5)
1991-2001	48,933 (33.0)
2002-2010	69,241 (46.8)
2011-2017	23,319 (15.7)
Age at arrival to Canada, mean (SD), y	23.7 (7.4)
Age category at arrival to Canada, y	
Infancy-9	7941 (5.4)
10-19	28,182 (19.0)
20-29	80,033 (54.0)
30-39	31,272 (21.1)
40-50	657 (0.4)
Canadian language ability at arrival to Canada	
English and/or French	92,177 (62.2)
Neither	55,851 (37.7)
Missing	57 (0.04)
Highest level of education at arrival to Canada	
Secondary school or less	78,864 (53.3)
Trade, no university or some university	22,289 (15.1)
University degree	35,410 (23.9)
Graduate degree	11,017 (7.4)
Missing	505 (0.3)
Immigration class at arrival to Canada	
Economic[a]	52,191 (35.2)
Sponsored family[b]	95,894 (64.8)
Duration of residence in Canada at index birth, y	
<10	111,651 (75.4)
≥ 10	36,434 (24.6)

[a]An immigrant woman or her family member (if she was a child) was selected for their skills and ability to contribute to Canada's economy.
[b]An immigrant woman or her family member (if she was a child) was sponsored by a Canadian citizen or a permanent resident living in Canada who is aged ≥ 18 years old.

© 2023 Jairam JA et al. *JAMA Network Open.*

eTable 4. Risk of Severe Maternal Morbidity (SMM) or Maternal Mortality Arising in the Index Delivery Hospitalization or up to 42 Days Thereafter, and the Top-20 SMM Indicators. Data are separately shown for immigrant and non-immigrant women residing in the lowest income quintile urban neighbourhood, and are limited to those who had a singleton hospital livebirth or stillbirth at $20^{0/7}$ to $42^{0/7}$ weeks' gestation in Ontario, Canada, 2002 to 2019.

**Immigrant women** (N = 148,085)[a]	**No. of events**	**Rate per 1000 births**		**Non-immigrant women** (N = 266,252)	**No. of events**	**Rate per 1000 births**
SMM or maternal mortality	2459	16.6		***SMM or maternal mortality***	4563	17.1
Maternal mortality, with or without concomitant SMM	13	0.1		***Maternal mortality, with or without concomitant SMM***	19	0.1
Top 20 SMM indicators				***Top 20 SMM indicators***		
Postpartum hemorrhage with red cell transfusion, or procedures to the uterus or hysterectomy	608	4.1		Postpartum hemorrhage with red cell transfusion, or procedures to the uterus or hysterectomy	1216	4.6
ICU admission	524	3.5		ICU admission	832	3.1
Puerperal sepsis	409	2.8		Puerperal sepsis	815	3.1
Hysterectomy	317	2.1		Severe preeclampsia & HELLP syndrome	560	2.1
Severe preeclampsia & HELLP syndrome	246	1.7		Curettage with red cell transfusion	315	1.2
Cardiac conditions	187	1.3		Hysterectomy	314	1.2
Eclampsia	160	0.1		Cardiac conditions	283	1.1
Curettage with red cell transfusion	131	0.9		Eclampsia	263	1.0
Procedures to the uterus or pelvic vessels with red cell transfusion	123	0.8		Assisted ventilation through endotracheal tube	215	0.8
Assisted ventilation through endotracheal tube	123	0.8		Complications of obstetric surgery & procedures	213	0.8
Repair of bladder, urethra, or intestine	101	0.7		Procedures to the uterus or pelvic vessels with red cell transfusion	197	0.7
Complications of obstetric surgery & procedures	99	0.7		Repair of bladder, urethra, or intestine	194	0.7
Acute abdomen	75	0.5		Obstetric embolism	125	0.5
Acute renal failure	57	0.4		Acute abdomen	112	0.4
Obstetric shock	52	0.4		Acute renal failure	106	0.4
Obstetric embolism	38	0.3		Obstetric shock	72	0.3
Intrapartum hemorrhage with red cell transfusion	33	0.2		Intrapartum hemorrhage with red cell transfusion	58	0.2
Placental abruption with coagulation defect	28	0.2		Septicemia during labour	55	0.2
Cerebrovascular diseases: subarachnoid & intracranial hemorrhage, cerebral infarction, stroke	27	0.2		Cerebrovascular diseases: subarachnoid & intracranial hemorrhage, cerebral infarction, stroke	44	0.2
Septicemia during labour	25	0.2		Placental abruption with coagulation defect	43	0.2

[a]Excludes refugee immigrants.

© 2023 Jairam JA et al. *JAMA Network Open.*

eTable 5. Risk of Severe Maternal Morbidity or Maternal Mortality Arising in the Index Delivery Hospitalization or up to 42 Days Thereafter, Comparing Immigrants From 10 Countries With the Greatest Number of Births in Ontario to Canadian-Born Women. Data are limited to women residing in the lowest income quintile urban neighbourhood, and who had a singleton hospital livebirth or stillbirth at $20^{0/7}$ to $42^{0/7}$ weeks' gestation in Ontario, Canada, 2002 to 2019.

Maternal country of birth exposure group[a]	No. (rate per 1000 births)	Unadjusted relative risk (95% CI)[b]	Adjusted relative risk (95% CI)[b,c]	Adjusted absolute risk difference per 1000 births (95% CI)[b,c]
Canadian-born (N = 266,252)	4563 (17.1)	1.00 (Reference)	1.00 (Reference)	0.0 (Reference)
Sri Lanka (N = 7800)	84 (10.8)	0.63 (0.51 to 0.78)	0.61 (0.49 to 0.76)	-7.5 (-9.6 to -5.5)
China (N = 13,080)	163 (12.5)	0.73 (0.62 to 0.85)	0.64 (0.55 to 0.75)	-5.9 (-8.0 to -3.8)
India (N = 21,129)	270 (12.8)	0.74 (0.66 to 0.84)	0.74 (0.65 to 0.83)	-4.5 (-6.1 to -3.0)
Pakistan (N = 15,166)	230 (15.2)	0.88 (0.78 to 1.01)	0.91 (0.80 to 1.04)	-1.4 (-3.4 to 0.6)
Vietnam (N = 3739)	59 (15.8)	0.92 (0.72 to 1.19)	0.86 (0.67 to 1.11)	-2.6 (-6.5 to 1.2)
Guyana (N = 3539)	59 (16.7)	0.97 (0.75 to 1.26)	0.96 (0.74 to 1.24)	-0.6 (-4.9 to 3.7)
Philippines (N = 15,084)	321 (21.3)	1.24 (1.11 to 1.39)	1.05 (0.94 to 1.18)	0.8 (-1.5 to 3.1)
Bangladesh (N = 5156)	126 (24.4)	1.43 (1.20 to 1.70)	1.43 (1.20 to 1.70)	6.7 (2.5 to 10.9)
Jamaica (N = 7974)	203 (25.5)	1.49 (1.29 to 1.71)	1.50 (1.30 to 1.73)	8.0 (4.5 to 11.5)
Ghana (N = 2733)	79 (28.9)	1.69 (1.36 to 2.11)	1.67 (1.34 to 2.08)	11.0 (4.8 to 17.1)

[a]Excludes 38 women missing country of birth, and 52,647 women not originating from the 10 countries with the greatest number of births in Ontario from 2002 to 2019.

[b]Using modified Poisson regression with a robust error variance. Generalized estimating equations with an exchangeable correlation structure accounted for correlated errors due to potentially more than one birth clustered within the same mother.

[c]Adjusted for maternal age (15-19, 20-29, 30-39, 40-50 years) and parity (0, 1, 2, ≥ 3).

© 2023 Jairam JA et al. *JAMA Network Open.*
