# Supplementary material for: Severe Maternal Morbidity and Mortality Among Immigrant and Canadian-Born Women Residing Within Low-Income Neighborhoods in Ontario, Canada
Source: JAMA Netw Open. 2023 Feb 16;6(2):e2256203. doi: 10.1001/jamanetworkopen.2022.56203 (PMC9936351; doi:10.1001/jamanetworkopen.2022.56203)
Supplement: Supplement 2. — Data Sharing Statement [file jamanetwopen-e2256203-s002.pdf]

Data Sharing Statement

Jairam. Severe Maternal Morbidity and Mortality Among Immigrant and Canadian-Born Women Residing Within Low-Income Neighborhoods in Ontario, Canada. *JAMA Netw Open*. Published February 16, 2023. doi:10.1001/jamanetworkopen.2022.56203

Data
Data available: No

Additional Information
Explanation for why data not available: The dataset from this study is held securely in coded form at ICES. While data sharing agreements prohibit ICES from making the dataset publicly available, access may be granted to those who meet pre-specified criteria for confidential access, available at https://www.ices.on.ca/DAS. The full dataset creation plan and underlying analytic code are available from the authors upon request, understanding that the computer programs may rely upon coding templates or macros that are unique to ICES and are therefore either inaccessible or may require modification.
